# MALDI-TOF MS Characterisation of the Serum Proteomic Profile in Insulin-Resistant Normal-Weight Individuals

**DOI:** 10.3390/nu13113853

**Published:** 2021-10-28

**Authors:** Katarzyna Pastusiak, Eliza Matuszewska, Dagmara Pietkiewicz, Jan Matysiak, Pawel Bogdanski

**Affiliations:** 1 Department of Treatment of Obesity, Metabolic Disorders and Clinical Dietetics, Poznan University of Medical Sciences, 84 Szamarzewskiego Street, 61-701 Poznan, Poland; pbogdanski@ump.edu.pl; 2 Department of Inorganic and Analytical Chemistry, Poznan University of Medical Sciences, 6 Grunwaldzka Street, 60-780 Poznan, Poland; eliza.matuszewska@ump.edu.pl (E.M.); dagmarapietkiewicz3@gmail.com (D.P.); jmatysiak@ump.edu.pl (J.M.)

**Keywords:** insulin resistance, normal-weight obesity, MALDI, protein-peptide profiling

## Abstract

Insulin resistance (IR) is one of the most common metabolic disorders worldwide and is involved in the development of diseases, such as diabetes and cardiovascular diseases, affecting civilisations. The possibility of understanding the molecular mechanism and searching for new biomarkers useful in assessing IR can be achieved through modern research techniques such as proteomics. This study assessed the protein–peptide profile among normal-weight patients with IR to understand the mechanisms and to define new risk biomarkers. The research involved 21 IR and 43 healthy, normal-weight individuals, aged 19–65. Serum proteomic patterns were obtained using matrix-assisted laser desorption/ionisation time-of-flight mass spectrometry. The proposed methodology identified six proteins differentiating normal weight IR and insulin sensitive individuals. They were fibrinogen alpha chain, serum albumin, kininogen-1, complement C3, serotransferrin, and Ig gamma-1 chain, which could potentially be related to inflammation. However, further investigation is required to confirm their correlation with IR.

## 1. Introduction

Insulin resistance (IR) is one of the most common metabolic disorders worldwide that can be defined as an impaired biological response to exogenous and endogenous insulin. It can disturb carbohydrate, lipid, and protein metabolism, as well as the mitogenic process [1]. It is primarily caused by disturbances in post-receptor hormone signalling and is observed in fatty tissues [2]. The adipose tissue is characterised by increased lipolytic activity and the release of large amounts of free fatty acids (PUFAs) that inhibit the action of insulin. Moreover, hypertrophic adipocytes are a source of pro-inflammatory cytokines that enhance IR within the adipose tissue, as well as in other tissues of the body [3]. Although IR is typically observed in obese people, the data show that approximately 21.34% of Polish women and 29.58% of Polish men with a normal body weight manifest impaired insulin sensitivity [4]. IR plays a key role in the development of metabolic syndrome as well as type 2 diabetes (T2D) [5]. Moreover, IR promotes atherosclerotic lesions and is an independent risk factor for cardiovascular disease [6]. Abnormal insulin sensitivity may also accompany other endocrinopathies, such as polycystic ovary syndrome (PCOS), hyperthyroidism, hyperprolactinemia, or hypercortisolemia [7]. In addition, it is associated with non-alcoholic fatty liver disease (NAFLD) and non-alcoholic steatohepatitis (NASH) [8]. The ‘gold standard’ in IR diagnostics is the hyperinsulinemia euglycemic clamp. However, in usual clinical practice involving indirect methods, the basis of fasting glucose and insulinemia are used, for example, Homeostatic Model Assessment of Insulin Resistance (HOMA-IR). The HOMA-IR values may be influenced by many factors; therefore, it is difficult to determine an appropriate cut-off point [9]. For the Polish population with normal glucose tolerance and a body mass index (BMI) <25 kg/m^2^, the value was determined to be above 2.1 [10]. Understanding the molecular mechanism and searching new biomarkers useful in assessing IR can be achieved through modern research techniques such as proteomics. Proteomics is a novel large-scale protein study that comprises proteomic analysis of the overall level of protein composition, structure, and activity. The genome remains stable throughout life and is identical for all organism cells, whereas the proteome could provide information regarding the impact of environment and other stimuli on a particular organism or cell. By applying this method for protein–peptide profiling and identification of distinctive proteins, it is possible to identify early disease biomarkers [11]. All these techniques provide the basis for proper prevention and treatment for this group of patients, which would undoubtedly lead to a decreased prevalence of metabolic syndrome and its complications. Previous research had analysed proteins in insulin-resistant obese and diabetic subjects and reported that the serum profiles differ in both humans and animals (i.e., mice) [12]. The same results were observed in a study on IR in patients with polycystic ovary syndrome [13]. This study assessed the protein–peptide profile among normal-weight patients with IR to understand the mechanisms and define new risk biomarkers. To the best of our knowledge, this is the first study to analyse protein–peptide profile in this group.

## 2. Materials and Methods

### 2.1. Study Groups

The protocol of this study was approved by the Bioethical Commission of Poznan University of Medical Sciences (approval no. 579/18). The participants had to meet the following inclusion criteria: age 19–65 years old, Caucasian race, BMI 18.5–25.0 kg/m^2^ and stable body mass 3 months prior to the study. The exclusion criteria were as follows: diabetes, polycystic ovary syndrome, clinical signs of hepatic, renal, adrenal or thyroid diseases; manifestation of neoplastic and autoimmune diseases; acute infection few months prior to the study; pregnant or breastfeeding; alcohol or drug abuse; currently smoking; using metformin or other drugs to improve insulin sensitivity and changes in diet three months prior to the study.

All participants received oral and written information about the research, and an informed consent form was signed after an explanation. Venous blood samples were collected from all participants from a cubital vein. The samples were incubated, centrifuged, and frozen at −80 °C. IR was calculated using Matthew’s method: fasting insulin (µIU/mL) × fasting glucose (mg/dL)/22.5 [14]. The cut-off points used for IR were >2.1. The general characteristics of IR and healthy subjects are presented in Table 1.

### 2.2. Serum Samples Pretreatment

The analyses were preceded by the purification and concentration of the serum samples. ZipTip C18 (Millipore, Bedford, MA, USA) reversed-phase chromatography micropipette tips were used for sample pre-treatment according to the manufacturer’s protocol (Millipore, Bedford, MA, USA). Briefly, serum samples were mixed with 0.1% trifluoroacetic acid (TFA) in a 1:5 ratio. Acetonitrile (ACN) and 0.1% TFA were used for tip conditioning. Afterward, the mixtures of serum samples and 0.1% TFA were loaded onto the ZipTip tips. Washing was performed using 0.1% TFA, and subsequently, bound peptides were eluted with 50% ACN in 0.1% TFA.

### 2.3. MALDI-TOF Proteomic Profiling

For MALDI-TOF-MS protein–peptide profiling, 1 μL each of the pre-treated samples was manually spotted onto an AnchorChip Standard Target Plate (Bruker Daltonics, Bremen, Germany) in triplicate. Drops of spotted samples were left to crystallise at room temperature (~23 °C). Each spot was covered with 1μL of the matrix solution (1.4 mg/L solution of alpha-cyano-4-hydroxycinnamic acid (HCCA) in a mixture containing 85% ACN, 15% water, 0.1% TFA, and 1 mM ammonium phosphate). MS analyses were performed using an UltrafleXtreme (Bruker Daltonics, Bremen, Germany) mass spectrometer in linear positive mode. Ions were detected in the range of *m*/*z* 1000–10,000. A total of 2000 laser shots were accumulated to acquire one spectrum for each spot. External calibration was performed using a mixture of the peptide calibration standard and protein calibration standard I (1:5, *v*/*v*). The average mass deviation from the reference mass was <100 ppm.

### 2.4. Data Analysis

Basic analyses were performed using Dell Statistica (data analysis software sys-tem), version 13.software (Dell Inc., Round Rock, TX, USA, 2016). A normality test was conducted with the Shapiro–Wilk test. Comparative analysis between groups for age and BMI was done using Student’s *t*-test for independent variables (normal distribution). Group differ-ences for glucose, insulin, and HOMA-IR were verified using the nonparametric Mann–Whitney U test (lack of normality). Due to the lack of normality, the correlations between MALDI-TOF MS spectra of serum samples and HOMA-IR were analysed us-ing the Spearman’s correlation coefficient test. A *p*-value of less than 0.05 was treated as significant.

The analysis of the recorded spectra was performed using ClinProTools software (version 3.0; Bruker Daltonics, Bremen, Germany). Multivariate classification algorithms (genetic algorithm (GA), supervised neural network (SNN), and quick classifier (QC)) were used for the model analysis. GA mimics the evolutionary process and is used for the selection of the most important variables for separation. The advantage of the GA is that much less computational time is needed than the brute force approach while still yielding good results. The main drawback is that only a near optimal solution can be obtain since all combinations of peaks should be tested to guarantee finding the best combination. QC is a univariate sorting algorithm. It calculates the average area of each peak. The averages are stored in the model with some statistical data like the *p*-values at certain peak positions. For classification, the peak areas are sorted per peak and a weighted average over all peaks is calculated. The univariate approach used in this algorithm makes it easy to trace back classification results. With only few samples available for the model generation, the validity of the classification seems to be better in comparison to other algorithms in many cases. SNN is a prototype-based classification algorithm that allows for an identification of the most characteristic spectra for each class. These spectra are called prototypes and are considered as prototypical samples of each class. The advantage of this algorithm is that it determines local classifier models (same class label may be connected to the different regions of prototypes), so it shows good performance for very multimodal data. For this algorithm it is also possible to determine a peak ranking. The main drawback is that it aims on empirical risk minimization which may stick in local minima [15]. The standard workflow for the statistical analyses has been described previously [16,17] and the analyses indicated differentiating peaks.

### 2.5. NanoLC-MALDI-TOF/TOF MS Identification of the Discriminative Peaks

The detailed procedure for identifying the discriminative peaks using an UltrafleXtreme (Bruker Daltonics, Bremen, Germany) tandem mass spectrometer has been described previously [18]. Briefly, before the analyses were conducted, the serum samples were purified using ZipTip C18 tips. The obtained eluates (4 µL) were subjected to nanoLC separation, automatically mixed with HCCA matrix solution, and spotted onto an AnchorChip 384 MALDI target plate (Bruker Daltonics, Bremen, Germany). The experiments were performed in the reflectron positive ion mode of the mass spectrometer in the mass range of *m*/*z* 700–3500. Protein–peptide identification was based on the SwissProt database and Mascot 2.4.1 search engine. The database searches were taxonomically restricted to *Homo sapiens.*

## 3. Results

**Figure 1:**
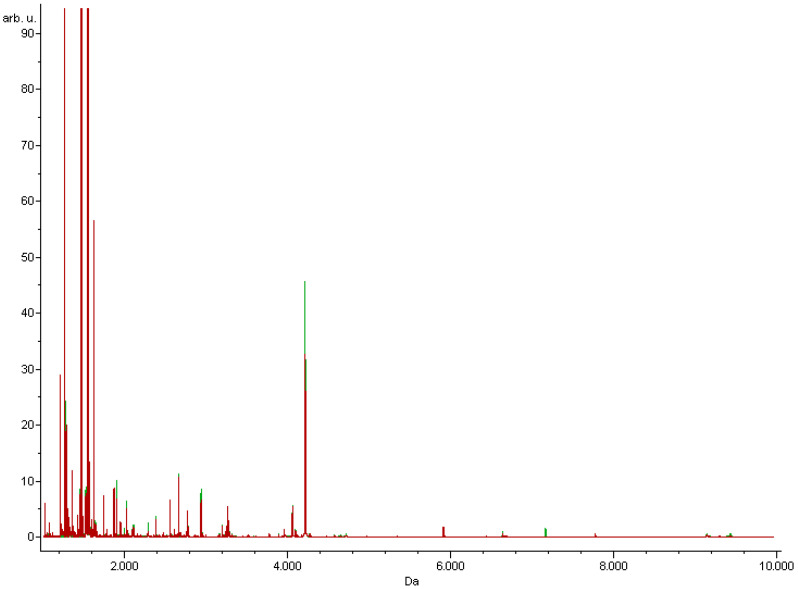
Total average MALDI-TOF MS spectra of serum samples derived from study participants. The spectra of insulin-resistant patients (red) and insulin- sensitive patients (green) are presented over the full scan range of *m*/*z* 1000–10,000.

The total average spectra of the IR and insulin-sensitive groups are shown in Figure 1. The data obtained from the MS analyses were statistically calculated using chemometric algorithms: a genetic algorithm, quick classifier, and supervised neural network. The differentiating peaks for each of algorithms are presented in Table 2. Some of the peaks are present in both the genetic algorithm and quick classifier, namely *m*/*z*: 1207.2 and 1305. The highest value of average cross-validation (80.56%), which determines the reliability of the algorithm-based model, was determined using a supervised neural network. The highest recognition capability (90.72%), indicating the percentage of individuals that are accurately assigned to the studied groups, was obtained using the genetic algorithm (Table 3). Recognition capability is expressed by the percentage of people covered by the study correctly assigned to the study and control groups. Cross validation is a measure for the reliability of a calculated model and can be used to predict how a model will behave in the future. Correct Classified parameter is a percentage of correctly classified part of valid spectra per class [15]. The different cross-validation and external validation values obtained using each chemometric algorithm are due to the differences in the mechanisms of the algorithms. The detailed basics of the algorithms are described in the literature [17]. Presumably, the most reliable method for data analysis in this study was genetic algorithm for which the highest external validation values were obtained.

The methodology used in this study identified a list of features which could differentiate between insulin- resistant and insulin-sensitive subjects. The fragmentation of the precursor ion *m/z* 1321.27 resulted in the peptide sequence K.STSGGTAALGCLVK.D with a high score in the Mascot database referring to Ig gamma-1 chain (IGHG1_HUMAN). Another identified protein is the fibrinogen alpha chain (FIBA_HUMAN) at the peaks at *m*/*z* 1546.37, 1207.20, 1077.93, 1020.86, and 1350.78 with the peptide sequences: A.DSGEGDFLAEGGGVR.G, G.EGDFLAEGGGVR.G, E.GDFLAEGGGVR.G, G.DFLAEGGGVR.G, and D.SGEGDFLAEGGGV.R. The fragmentation of signal *m*/*z* 1305.22 allowed the identification of the following sequence: K.ECCEKPLLEK.S, which gave a significant score in the Mascot search for serum albumin (ALBU_HUMAN). Analysis also revealed a peak at *m*/*z* 1568.41 with the peptide sequence H.GHEQQHGLGHGHKF.K. It gave a significant score in the Mascot search for Kininogen-1 (KNG1-HUMAN). Another identified protein was complement C3 (CO3_HUMAN), with peaks at *m*/*z* 1519.79 and 2755.13, with the peptide sequences G. SPMYSIITPNILR. L and R.EGVQKEDIPPADLSDQVPDTESETR.I. Moreover, the analysis of the precursor ion *m*/*z* 1283.24 resulted in the identification of serotransferrin (TRFE_HUMAN) with significant Mascot hits based on the peptide fragmentation sequence K.EGYYGYTGAFR.C. The data for the identified proteins are summarised in Table 4.

NanoLC-MALDI-TOF/TOF MS/MS analyses were conducted in the mass range of *m*/*z* 700–3500. Therefore, the peaks of *m*/*z* 3964.52, 9133.97, 8916.44, 4122.57, and 3996.08 could not be identified. Moreover, some precursor ions with masses below *m*/*z* 3500 could not be identified, likely due to the presence of neighbouring peaks and insufficient resolution. These precursors require further investigation.

The associations between MALDI-TOF MS spectra of serum samples and HOMA-IR are presented in Table 5. There were no significant relationships between MALDI-TOF MS spectra and HOMA-IR in research and control group as well as overall.

## 4. Discussion

This study aimed to assess the protein–peptide profile among normal-weight patients with IR to understand the mechanisms and define new risk biomarkers. Currently, the diagnostic criteria for IR are based on biochemical parameters such as glucose and insulin level [9]. We suggest that exploring the molecular mechanism of IR development will contribute to its early prevention, while preventing the development of metabolic syndrome, cardiovascular diseases, and the associated complications. The proposed methodology identified six proteins differentiating normal weight IR and insulin sensitive individuals. They were fibrinogen alpha chain, serum albumin, kininogen-1, complement C3, serotransferrin, and Ig gamma-1 chain. Fibrinogen plays a key role in fibrin formation, which is one of the components of blood clots. Innate- and T cell-mediated pathways may also facilitate the immune response [19]. A number of studies have linked plasma fibrinogen with different components of metabolic syndrome. Previous research has revealed a strong association between fibrinogen and IR in a non-diabetic group of patients. These relationships remained significant after adjusting for BMI [20]. One of the main mechanisms is development of IR inflammation [3]. Fibrinogen was identified as one of the inflammation-sensitive plasma proteins (ISPs), positively correlated with body fatty tissue and inflammatory markers [21]. Therefore, the elevated levels of fibrinogen in IR subjects could be a reaction to inflammatory conditions and excess production of ISPs. Serum albumin is another recognised protein, and its major role is the regulation of the colloidal osmotic pressure of blood and mineral transportation in the body [22]. Previous research has confirmed the positive correlation between serum albumin level and metabolic syndrome [23]. A Korean study on non-diabetic individuals revealed that serum albumin concentration was associated with higher levels of HOMA-IR. However, it did not have a protective effect against incident diabetes [24]. The causal relationship between IR and serum albumin level is unclear. Some results indicate a reverse link and suggest that hyperinsulinemia caused by IR may affect serum albumin levels [25,26]. Kininogen-1 is an inhibitor of thiol proteases, which are responsible for the production of kininogens. Kininogens can be high molecular weight and low molecular weight, both of which are part of the plasma kallikrein–kinin system [27]. This system is activated in response to many physiological and pathophysiological conditions, including blood coagulation, regulation of blood pressure, pain, and inflammation [28]. Kininogen-1 has recently been investigated as a biomarker in cancer and mental diseases [29,30,31]. However, there are no data regarding its relationship with IR. The only potential pathway connecting this protein with IR could be its role in inflammation. Component C3 is another protein that plays an essential role in the activation of the complement system [32]. Numerous studies have confirmed the relationship between component C3 and IR in different groups of patients: non-diabetic [33], obese [34], and psoriatic arthritis patients [35]. A Chinese study on women with metabolic syndrome revealed that C3 might be a stronger inflammatory marker of IR than high-sensitivity C-reactive protein [36]. Moreover, in research on normal-weight obese women, a positive correlation between component C3 and IR was observed [37], This suggests that the C3 concentration could be mostly dependent on the percentage of fatty tissue rather than body weight. Serotransferrin, an iron-binding transport protein, was also identified during this study [38]. No previous studies have reported a correlation between serum serotransferrin concentration and IR. However, there are some reports about the role of iron and transferrin in inflammation and IR development [39,40,41]. The pathway could potentially link serotransferrin with IR, which would indicate that iron plays a role in oxidative stress processes. The last identified protein was the Ig gamma-1 chain, which participates in the immune process [42]. In the literature, there are no data about the correlation between the Ig gamma-1 chain and the pathogenesis of IR.

In our study, a direct relationship between the identified proteins and insulin resistance has not been confirmed. This may mean that differences in the protein-peptide profile of normal weight subjects with and without insulin resistance result from other variables. However, the limitation of the study is a relatively small research group, so future studies involving a larger group are necessary to verify these results.

## 5. Conclusions

In summary, the majority of identified proteins could take part in the inflammation process. However, the study did not confirm a direct relationship between them and insulin resistance. Further extensive analysis is required to verify the presence of associations. The biological role of proteins in IR should be explored because such studies have the potential to have a useful impact on the development of diagnostic and therapeutic strategies.

## Figures and Tables

**Table 1:** Characteristics of the groups.

Parameters	Research Group	Control Group	*p*-Value
Numbers	21	43	
Men	4	13	0.3858
Women	17	30	
Age [years]	44.10 ± 13.26	43.26 ± 12.26	0.8426
BMI [kg/m^2^]	22.18 ± 2.27	22.61 ± 2.04	0.2671
Glucose [mg/dL]	96.38 ± 17.87	87.09 ± 12.85	<0.0001
Insulin [mmol/L]	21.13 ± 9.93	6.91 ± 3.95	0.0015
HOMA-IR	4.99 ± 2.07	1.54 ± 1.23	<0.0001

BMI—Body mass index, HOMA-IR—Homeostatic Model Assessment of Insulin Resistance.

**Table 2:** Peaks discriminating study groups generated by the applied algorithms.

GA	QC	SNN
1321.27 3240.65 1546.37 3964.52 1305.22 1207.20 1897.25 3278.35 1077.93 9133.97 1568.41 1519.79 8916.44 4122.57 3996.08	1020.86 1207.20 1269.76 1283.24 1305.22 1350.78	2755.13

GA—genetic algorithm, QC—quick classifier, SNN—supervised neural network.

**Table 3:** Values of chemometric parameters for the algorithms.

	GA	QC	SNN
Cross-validation (%)	44.15	57.6	80.56
Recognition capability (%)	90.72	56.51	46.42
Correct classified (%)			
Insulin resistance	71.4	14.3	92.9
Control	96.3	100	0

GA—genetic algorithm, QC—quick classifier, SNN—supervised neural network.

**Table 4:** List of identified proteins discriminating between insulin-resistant patients and control group.

No.	Precursor Ion *m*/*z*	Peptide Fragmentation Sequence	UniProtKB-ID	Protein Name
1.	1321.27	K.STSGGTAALGCLVK.D	IGHG1_HUMAN	Ig gamma-1 chain
2.	1546.37	A.DSGEGDFLAEGGGVR.G	FIBA_HUMAN	Fibrinogen alpha chain
	1207.20	G.EGDFLAEGGGVR.G		
	1077.93	E.GDFLAEGGGVR.G		
	1020.86	G.DFLAEGGGVR.G		
	1350.78	D.SGEGDFLAEGGGV.R		
3.	1305.22	K.ECCEKPLLEK.S	ALBU_HUMAN	Serum albumin
4.	1568.41	H.GHEQQHGLGHGHKF.K	KNG1-HUMAN	Kininogen-1
5.	1519.79	G.SPMYSIITPNILR.L	CO3_HUMAN	Complement C3
	2755.13	R.EGVQKEDIPPADLSDQVPDTESETR.I		
6.	1283.24	K.EGYYGYTGAFR.C	TRFE_HUMAN	Serotransferrin

**Table 5:** Corelation between MALDI-TOF MS spectra of serum samples and HOMA-IR.

Protein Name	Precursor Ion *m*/*z*	*Spearmana’s* R-Value			*p*-Value
		Overall	Research Group	Control Group	
Ig gamma-1 chain	1321.27	−0.17	−0.17	−0.12	statistically insignificant >0.05
Fibrinogen alpha chain	1020.86	0.06	−0.04	−0.07	
	1077.93	0.04	−0.09	0.19	
	1207.20	0.16	−0.09	0.15	
	1350.78	−0.04	−0.24	−0.17	
	1546.37	−0.16	−0.39	0.13	
Serum albumin	1305.22	−0.14	−0.20	−0.11	
Kininogen-1	1568.41	−0.14	−0.28	−0.16	
Complement C3	1519.79	−0.14	−0.28	0.09	
	2755.13	0.13	−0.46	0.34	
Serotransferrin	1283.24	−0.09	−0.26	−0.31	

## Data Availability

The data presented in this study are available on request from the corresponding author.
